# The Characteristics and Effectiveness of Oral Healthcare Education Interventions for Stroke Clinicians: A Scoping Review

**DOI:** 10.1111/jocn.17795

**Published:** 2025-04-28

**Authors:** Ajesh George, Lien Lombardo, Shilpi Ajwani, Rochelle Wynne, Paula Sanchez, Ariana Kong, Bhavya Talluri, Scott William, Caleb Ferguson

**Affiliations:** ^1^ Australian Centre for Integration of Oral Health (ACIOH), School of Nursing & Midwifery Western Sydney University Liverpool New South Wales Australia; ^2^ Ingham Institute for Applied Medical Research Liverpool New South Wales Australia; ^3^ School of Nursing University of Wollongong Wollongong New South Wales Australia; ^4^ Sydney Dental School Faculty of Medicine and Health, University of Sydney Surry Hills New South Wales Australia; ^5^ Sydney Dental Hospital, Oral Health Services, Sydney Local Health District Surry Hills New South Wales Australia; ^6^ School of Nursing & Midwifery Deakin University & Western Health Melbourne Victoria Australia; ^7^ Centre for Chronic & Complex Care Research, Blacktown Hospital Western Sydney Local Health District Blacktown New South Wales Australia

**Keywords:** attitude, confidence, implementation, knowledge, oral health, practice, scoping review, stroke

## Abstract

**Aims:**

To explore the characteristics of oral healthcare education interventions for stroke clinicians and the effectiveness of these interventions in improving the oral health knowledge, attitudes, confidence, and practice among acute stroke clinicians.

**Design:**

Scoping review, guided by Arksey and O'Malley's (2005) framework.

**Methods:**

Original full‐text studies reporting educational oral healthcare interventions for stroke clinicians, including but not limited to nurses, were eligible for inclusion. Included studies were extracted and appraised using the Joanna Briggs Institute (JBI) Checklist aligned to the study methodology. Narrative synthesis was used to describe heterogeneous findings.

**Data Sources:**

Key electronic bibliographic databases including CINAHL, Cochrane, MEDLINE (Ovid), ProQuest, Pubmed, and Scopus, in addition to grey literature, were searched for studies published between 1st January 2000 and 20th January 2024.

**Results:**

Five studies conducted in acute inpatient settings were included: two randomised controlled trials, two mixed‐methods studies, and one quality improvement project. Most (*n* = 4) studies developed complex interventions that included education and other components (products, referral pathways, assessment tools), and were delivered either face‐to‐face or as an online program. Most studies reported positive changes in oral health knowledge, attitudes, and confidence. There was limited measurement of the acceptability and feasibility of the interventions, with only one study reporting positive feedback from clinicians. There was no evidence to support changes in clinical practice following any of the included interventions.

**Conclusion:**

Existing evidence indicates interventions for stroke clinicians have some potential for building stroke clinicians' capacity to provide adequate oral healthcare. There is however no evidence linking these interventions to optimised patient outcomes. There is a need for research focused on the implementation and dissemination of tailored oral health educational interventions incorporating clinically meaningful outcomes.

**Implications for Profession/Patient Care:**

Existing oral healthcare educational interventions appear to have a positive effect on stroke clinicians' oral health knowledge, confidence, and attitudes. Educational interventions in oral healthcare are perceived to be acceptable and feasible; however, further research is needed to design and test the effect of new interventions.

**Impact:**

Integrated oral healthcare is particularly important for stroke survivors who are at greater risk of preventable aspiration pneumonia. This scoping review highlights the characteristics of existing educational programs for stroke clinicians, their effectiveness, and gaps in existing evidence. Review findings substantiate the need for future research to enhance existing oral healthcare interventions, to translate knowledge acquired from training into clinical practice, and to capture appropriate measures of effect.

**Reporting Method:**

The PRISMA‐ScR Checklist.

**Protocol Registration:**

This review was registered with the Open Science Framework registry (https://doi.org/10.17605/OSF.IO/4BWVF).

**Summary**

- What does this paper contribute to the wider global clinical community?
  ○ Oral healthcare educational interventions for stroke clinicians are typically tailored to the local context involving education with dental products, assessment tools, or referral pathways.
  ○ Stroke clinicians are receptive to oral healthcare educational interventions.
  ○ Further work is needed to explore how to effectively translate improvements in clinical oral health knowledge into practice.

## 1 Introduction

Stroke is a leading cause of death, disability, and global health burden with a reported 12.2 million cases and 6.5 million deaths annually. In 2019, 101 million people were currently living or had experienced stroke in their life, an increase of 85% from 2010 that equates to a 32% (143 million) increase in disability adjusted life years due to stroke, for the same time period (Feigin et al. 2021). The economic impact of stroke is estimated to be $USD65.5B, inclusive of direct (hospitalisation and rehabilitation) and indirect (loss of productivity) costs due to disability (Wentworth et al. 2009).

Up to half of stroke survivors experience cognitive or functional disability that impacts their activities of daily living (Carmo et al. 2015; Katan and Luft 2018). The inability to perform basic tasks like tooth brushing and flossing negatively impacts the oral health of stroke survivors (Kwok et al. 2015). Poor oral health results in oral cavity complications such as plaque, gingivitis leading to more serious periodontitis, increased bacteria colonisation, and infection (Ajwani et al. 2021; Lyons et al. 2018). Another major consequence of stroke is dysphagia, or difficulty with swallowing, which along with poor oral health can increase the risk of aspiration pneumonia (Ajwani et al. 2017; González‐Fernández et al. 2013; Shaker and Geenen 2011). Stroke‐associated pneumonia, often occurring within 7 days of a stroke event, is estimated to affect 10% of patients and is a leading cause of mortality and morbidity (Badve et al. 2019; Wagner et al. 2016). Due to post‐stroke risk of pneumonia and other complications such as malnutrition and dehydration, it is imperative that oral healthcare is prioritised in stroke care settings to help optimise speech, nutritional intake, systematic health, rehabilitation outcomes, and quality of life for individuals, and to reduce health system burden (Ajwani et al. 2017; Campbell et al. 2020; Chipps et al. 2014).

International stroke guidelines, across UK (Intercollegiate Stroke Working Party 2016), Australia (Stroke Foundation, 2022), and New Zealand (Stroke Foundation of New Zealand and New Zealand Guidelines Group 2010) all identify clinicians, including the nursing workforce, as key to providing optimal oral healthcare for stroke patients in the acute inpatient setting. However, existing evidence indicates clinicians are placing limited emphasis on oral healthcare during stroke rehabilitation (Ab Malik et al. 2018; Ajwani et al. 2017; Talbot et al. 2005). A large national survey conducted in Scotland involving 71 stroke units revealed a significant lack of standardisation in the methods employed for oral care across various facilities. Moreover, considerable variability was observed around access to staff training in oral health, assessments, protocols, and oral hygiene materials (Talbot et al. 2005) These inconsistencies in oral health care were reiterated in later studies including a scoping review where poor patient attitude and awareness along with limited oral health knowledge among stroke clinicians were identified as contributing factors (Ajwani et al. 2017; Ab Malik et al. 2018). These barriers are concerning as they may hinder the clinicians from delivering sufficient oral hygiene care (Talbot et al. 2005) When the perspectives of nursing and allied health stroke clinicians have been investigated, the need for an integrated oral healthcare model for stroke survivors has been supported (Ferguson et al. 2020). Clinician‐targeted oral healthcare educational interventions could be a key strategy in improving the oral health of stroke survivors.

There is growing evidence that robust, evidence‐based educational–behavioural interventions are required to improve clinician engagement and delivery of oral healthcare interventions (Ajwani et al. 2021, 2017; Ferguson et al. 2020; Kwok et al. 2015; Lyons et al. 2018). Such interventions could help improve the oral health knowledge and confidence of clinicians and their access to protocols and resources. To date, there is limited evidence describing the availability of clinician‐targeted oral healthcare educational interventions and, more importantly, a lack of comprehensive assessment of the effectiveness of existing interventions to improve oral healthcare provision for stroke survivors. Existing evidence has a limited focus on the assessment of protocols, education programmes and oral care products (Campbell et al. 2020; Gurgel‐Juarez et al. 2020). Therefore, there is a need to identify and evaluate suitable and effective educational interventions for stroke clinicians to support them in improving knowledge, adhering to practice guidelines and delivering optimal oral healthcare to their patients after stroke. The aim of this study is to address this gap in the evidence.

## 2 Aims

Formulating the aim of this review was guided by the Joanna Briggs Institute (JBI) participants, concept and context framework by Peters et al. (2020). The overarching aim is to explore current education interventions that improve stroke clinicians' oral healthcare management of stroke survivors in acute stroke and rehabilitation settings. Specific review objectives were to explore:

- The characteristics of current oral health education interventions for stroke clinicians
- The effectiveness of these educational interventions

## 3 Methods

### 3.1 Design

A scoping review was undertaken to map key concepts, main sources, and types of evidence to synthesise the current literature in an emerging research area (Tricco et al. 2016). This was appropriate as there is limited evidence on oral care education interventions targeted at stroke clinicians. Additionally, this design enabled a wider review of the topic area particularly around characteristics of existing educational interventions (which was important to inform future strategies), unlike systematic reviews which are hypothesis‐testing. Further, due to the limited evidence in this area, the iterative approach of a scoping review allowed the team to consider a broader range of grey literature involving unpublished resources like quality improvement projects. The Arksey and O'Malley (2005) framework, used to guide the methodological approach for the review, has five phases: (i) Identifying the research question, (ii) Identifying relevant studies, (iii) Study selection, (iv) Charting the data, and (v) Collating, summarising, and reporting the results. The Preferred Reporting Items for Systematic reviews and Meta‐Analysis extension for Scoping Reviews (PRISMA‐ScR) Checklist was used to guide the structure of this review (Tricco et al. 2018) (Data S1). The review was registered with the Open Science Framework (OSF) Registry (Registration DOI: https://doi.org/10.17605/OSF.IO/4BWVF).

### 3.2 Search Methods

A search of the following key electronic bibliographic databases was conducted with the assistance of a university health librarian in April 2022, and was updated in January 2024: CINAHL, Medline (Ovid) (1996‐current), ProQuest, PubMed, SCOPUS, and Cochrane library. Key search terms (stroke, mouthcare, oral care/dental health, clinicians/nurses/allied health, and education/training/promotion) were combined with Boolean operators (and/or), truncations, and MeSH (Medical subject headings) terms tailored to each database platform. Other sources including organisations, grey literature, and bibliographic hand searching of relevant sources were used to identify relevant literature. A detailed search strategy is included in Data S2.

### 3.3 Inclusion and/or Exclusion Criteria

#### 3.3.1 Population

Nursing, allied health, physicians, and other stroke care clinicians caring for patients after stroke.

#### 3.3.2 Concept

Educational interventions to improve stroke clinicians' oral care provision, including knowledge, attitudes, and practice. We excluded studies that involved interventions targeted to improve patients' oral care knowledge and practice.

#### 3.3.3 Context

Original research studies published between 1st January 2000 to 20th January 2024, reporting results of educational interventions. Any studies that had mixed‐population or non‐stroke specific settings, were considered if there was available data to extrapolate results specific to stroke‐care clinicians care of patients post‐stroke. Studies that were not in acute or rehabilitation stroke settings, such as nursing homes, home‐based chronic care, neurology, general medicine, intensive care, and community settings, were excluded. We excluded reviews, discussion papers, study protocols, editorials, commentary, and conference abstracts, as well as studies targeted to populations other than stroke, and those not published in the English language. Studies that did not have full text available were not included.

### 3.4 Selection Process

An initial search was conducted of databases and other sources by LL and exported into EndNote (Version 20.3) reference manager, to enable abstract and full text screening. After removing duplicates, studies were screened and excluded according to title and abstract. Studies deemed to be ‘unclear’ progressed to full text review to establish suitability. Full text review was conducted independently by two reviewers (LL & PS) and in the case of conflicting opinions, a third reviewer (AG) was included to reach consensus.

### 3.5 Quality Appraisal

To understand the rigour of the research and the quality of the interventions developed, a quality appraisal was undertaken. A quality appraisal would also strengthen the review's future recommendations for research, policy, and practice in this area (Pham et al. 2014). The quality of studies was assessed using the Joanna Briggs Institute (JBI) Checklist that aligned with the methodology of corresponding studies (Aromataris and Munn 2020). These included the JBI tools for quasi‐experimental studies and randomised control trials. Two separate investigators (AG, SW) scored the included studies by assigning 1 point for each applicable item. A third author (PS) was consulted to resolve any discrepancies. After reaching consensus among authors, cut‐off values were established including 0%–59% considered poor quality, 60%–79% considered moderate quality, and 80% or greater considered high quality (Goldsmith et al. 2007). No articles were excluded in this review based on quality appraisal.

### 3.6 Data Extraction

Data extraction and screening was undertaken by LL and PS, using a Microsoft Word extraction template that captured study characteristics including author names, year published, study location, study aims, design, setting, population, intervention characteristics, and main findings describing measures of effectiveness. Iterative discussions took place between LL, PS, and AG to enable refinement of extracted data prior to analysis. When extracted data required clarification, lead authors were contacted.

### 3.7 Data Synthesis

Due to the heterogeneity of included studies, a narrative synthesis and summary tables were utilised to synthesise the findings of this scoping review. Tabulation of results included (i) study characteristics, (ii) intervention characteristics, and (iii) intervention effectiveness according to Arksey and O'Malley's (2005) recommendations.

## 4 Results

**FIGURE 1:**
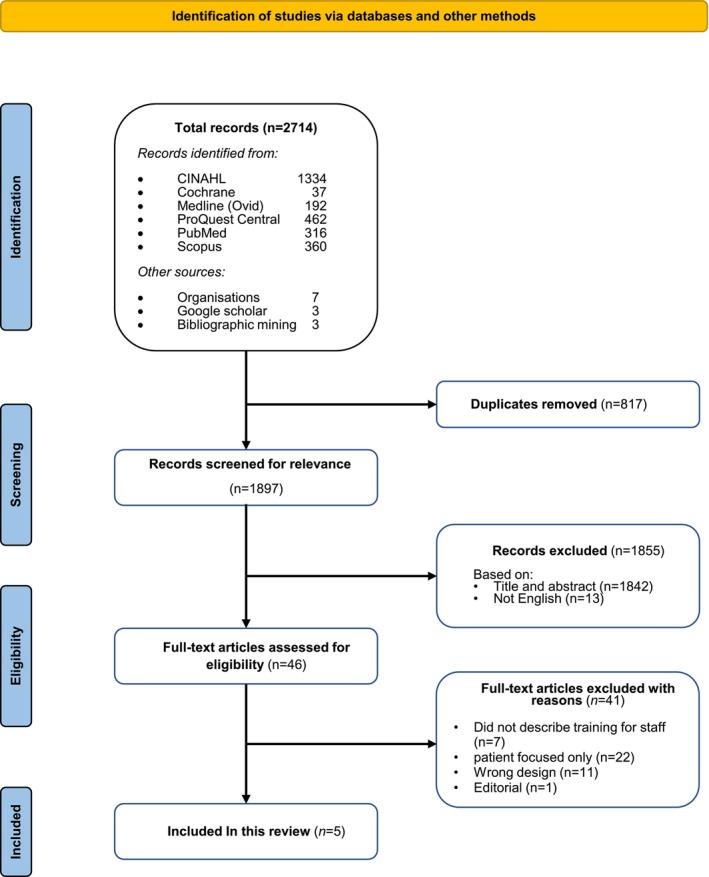
PRISMA flow diagram. [Colour figure can be viewed at wileyonlinelibrary.com]

A total of 2714 articles were returned after running the search across the databases and platforms. After removing 817 duplicates, 1897 records were screened. A total of 1855 records were excluded based on their title and abstract (*n* = 1842) and for being published in a language other than English (*n* = 13). The full text was retrieved for the remaining 46 studies, of which five were included in the final review. Patient rather than clinician‐focused interventions (*n* = 22) were the most frequent reason for exclusion. The results of the search are presented in a PRISMA flow diagram (Figure 1).

### 4.1 Study Characteristics

Study characteristics are summarised in Table 1. The five included studies (Ab Malik et al. 2017; Brady et al. 2011, 2020; Letsos et al. 2013; Smith et al. 2017) were published between 2005 and 2020. Three were from the UK (Brady et al. 2011, 2020; Smith et al. 2017), one from Canada (Letsos et al. 2013) and one from Malaysia (Ab Malik et al. 2017). A multisite randomised controlled trial (RCT) design was used by two studies (Ab Malik et al. 2017; Brady et al. 2020), a further two used mixed methods design (Brady et al. 2011; Smith et al. 2017) and there was a single prospective quality improvement project (Letsos et al. 2013). Studies were conducted in inpatient hospital settings, in dedicated stroke units caring for acute and/or rehabilitation stroke populations (Brady et al. 2011, 2020; Smith et al. 2017) or in mixed or general wards which cared for stroke patients (Ab Malik et al. 2017; Letsos et al. 2013). Four of the five studies were rated as moderate‐high quality, with only one study rated as poor (Letsos et al. 2013).

**TABLE 1:** Summaries of included studies.

Author, year, study location	Aims	Design	Setting and population	Intervention	Main study findings	Quality assessment
Ab Malik et al. (2017), Malaysia	To evaluate the effectiveness of a Web‐based continuing professional development (CPD) program on “general intention” of the health carers to perform daily mouth cleaning for stroke patients using the theory of planned behaviour (TPB)	Double‐blinded cluster randomised controlled trial	Registered Nurses caring for stroke patients, across 10 public hospitals in Malaysia (mainly from rehabilitation and general medical wards) (*n* = 547)	Intervention: Oral care provision specific Web Based CPD program, developed by stroke physicians and dentists Control Group & guided by theory of planned behaviour (TPB) Control: Analogous web based CPD program related to “bundles of care” for stroke patients, including some details to oral hygiene, not specific to TPB	The Web‐based CPD program based on TPB increased general intention, attitudes, subjective norms, and knowledge to provide oral hygiene care among stroke carers for their patients: 1‐month post‐intervention, there was a significant difference in changes in scores of attitude (*P* = 0.004) and subjective norm (*p* = 0.01), but not in other TPB domains (GI, *p* = 0.11; PBC, *p* = 0.51; or knowledge, *p* = 0.08) between test and control group At 6 months, there were significant differences in changes in scores of GI (*p* = 0.003), attitude (*p* = 0.009), SN (*p* < 0.001) and knowledge (*p* = 0.001) between test and control group	High
Brady et al. (2011), United Kingdom	To develop and evaluate the implementation of a complex, multidimensional OHC intervention for people in stroke rehabilitation settings which would inform the development of a future RCT	Mixed methods study	Nursing staff (including clinical support workers and students) in single mixed stroke care ward for acute stroke care and rehabilitation (*n* = 26). MDT also invited to participated in intervention	Complex OHC intervention of which clinician‐targeted education component included 2 h training package by specialists, across 8 training sessions on: OHC assessment OHC protocol OHC Equipment OHC Products	In the area of education effectiveness, attitudes and knowledge had most significant improvements, following training Acceptance of OHC protocol was not straightforward, with poor adherence to certain recommendations in protocol Despite allied health being invited to same training, nursing staff perceived little or no input from MDT post training Staff found guidance in protocol on care and cleaning of dentures helpful Training had positive benefits with clinician's own OHC practices, beyond patient care OHC assessment and protocol was well liked in terms of relevance in content and ease of use	High
Brady et al. (2020), United Kingdom	To pilot the delivery of enhanced Oral healthcare (OHC) across multiple stroke wards in a pragmatic, stepped‐wedge, cluster randomised controlled trail of the effectiveness of enhanced versus usual OHC	Pilot, pragmatic, stepped wedge, Cluster randomised controlled trail design	Nursing staff including Registered Nurses, assistants and students caring for stroke patients across 4 hospitals (*n* = 112)	Intervention was a co‐produced multicomponent enhanced OHC intervention of which the staff training course consisted of 90‐min online OHC training, facilitated by senior nurses which covered best practice OHC information, and OHC assessment and care tutorial. Training was optional There was also access to the following on wards: OHC assessment OHC protocol OHC equipment OHC products	Variability in training engagement from staff across the four sites (ranging from 41.1% to 94.7% of all staff) Majority of staff reported no change in OHC attitude and knowledge after training Poor staff adherence to documenting OHC assessment and plans, but increased slightly during OHC enhanced phase	High
Letsos et al. (2013), Canada	Implementation of a standardised oral care best practice initiative with 5 key components, of which education for interprofessional team was incorporated	Quality improvement project	Acute neurology & neurosurgery care inpatient setting (*n* = 46, 30% of staff)	Five component standardised oral care practice initiative rolled out across two units, of which interprofessional team education was a component. Education Intervention: 20‐min education session Hands‐on learning with recommended oral care tools and products	Post‐implementation bedside audit showed 96.4% compliance of Oral Health Assessment tool use by staff within 24 h of admission, compared to 47.8% for same time period pre‐implementation Oral care plans for patients with dysphagia clearly communicated, evidenced by interprofessional documentation in medical charts of practice Daily interprofessional collaboration occurring with regards to patient‐specific oral care needs and recommendations, as documented in patient's medical charts Improved stocking of recommended oral care products and located at bedside	Poor
Smith et al. (2017), UK	To develop an oral hygiene complex intervention and evaluate its feasibility in a single UK stroke Centre, which included staff education	Sequential mixed methods design	An Acute Stroke Unit (ASU) and Stroke Rehabilitation Unit (SRU) Staff‐ Nursing and Healthcare assistants (*n* = 13)	A oral hygiene complex intervention comprising: (i) web‐based education and ‘hands‐on’ practical training for stroke unit nursing staff, (ii) a pragmatic oral hygiene protocol consisting of twice‐daily powered (or manual if preferred) brushing with chlorhexidine gel (or non‐foaming toothpaste) ± denture care Education component included—A web‐based education programme comprising four discrete modules Followed by practical hands‐on training by oral care trained staff Followed by assessment of practical elements of protocol to be deemed competent	Focus group results found: Barriers to education included accessibility and interruptions on floor In terms of acceptability, staff found the education easy to use and helped their oral care knowledge, however may not reflect ‘real‐life experience’ In terms of adequacy, education provided increased knowledge levels, raised awareness of the need for mouthcare and built self‐confidence among staff	Moderate

### 4.2 Intervention Characteristics

Oral healthcare education interventions were generally delivered as part of complex interventions, targeted predominantly at the nursing workforce and often informed by either design principles, expert stakeholders, or a co‐design approach. There was heterogeneity in the format, delivery, and content of education programmes in addition to support material and resources employed. In four of five of the studies, oral healthcare education was implemented and evaluated as part of a complex intervention, accompanied by the delivery of other components including an oral healthcare protocol, assessment tool, oral healthcare products and equipment, and support staff (Brady et al. 2011, 2020; Letsos et al. 2013; Smith et al. 2017). A summary of intervention characteristics is provided in Table 2.

**TABLE 2:** OHC education intervention characteristics.

	Ab Malik et al. (2017)	Brady et al. (2011)	Brady et al. (2020)	Letsos et al. (2013)	Smith et al. (2017)
Intervention characteristics					
1.1 Was education part of complex intervention?	No	Yes	Yes	Yes	Yes
1.2 Recipients	Registered nurses (RNs) 373 of 547 participants responded to surveys	All nursing staff including RN's clinical support workers and students (*Multidisciplinary team were invited to attend training but not part of evaluation*) 26 Nursing staff participated in staff questionnaires	Of 123 Nursing staff employed (incl. RNs, clinical support workers and students—112 consented) 108—pre‐intervention survey 74—did training 75—post‐intervention survey 83—Enhanced Care phase survey	All multidisciplinary team (MDT) Unclear of total staff numbers Estimated at 153 staff members as 30% of workforce responded to pre‐intervention survey (*n* = 46)	Nurses and healthcare assistants All staff completed training (*n* = 50) and 13 participated in focus groups
1.3 Development principles/framework	Theory of Planned Behaviour (TPB) Content designed by rehabilitation medicine stroke physicians and dentists, following good practices of computer assisted Learning for oral health	No framework indicated Education designed by experienced specialist gerodontologist	Co‐design of complex intervention with stroke survivors, carers and stroke specialist MDT	Best practice guidelines and literature to inform the project design, including education component	Medical Research Council (MRC) framework for complex intervention design Co‐design with nursing staff and MDT
1.4 Content	Good oral healthcare Poor oral hygiene consequences Importance of Nurse's role and care of stroke patients	Ideal context and nature of supporting patient's OHC following stroke OHC protocol and assessment tools OHC products and equipment	Evidence‐based or best practice OHC information Tutorial on OHC assessment & OHC care	Oral care best practice OHC products and equipment (hands on training)	Four discrete modules covering anatomy and physiology, common problems on assessment and, videos showing administering OHC protocol to stroke patients OHC products and equipment (hands on training post‐online training)
1.5 Format and delivery	Web‐based non‐compulsory training	Face to face training by specialist All nursing staff received training	Web‐based ‘optional’ training	Face to face training given to all nursing and MDT staff Delivered by Clinical Neurological Sciences (CNS) clinical educator and Southwest Ontario Stroke Network regional education coordinator, with input from the Speech language pathologists (S‐LP)	Hybrid of web‐based and face to face training, followed by practical assessment of all nursing and HCA staff End of module assessment to progress Face to face training and practical assessment by specially trained “oral care link nurses” Simulation exercise as part of face to face training
1.6 Duration & frequency	Duration of intervention not reported Reminders every 6 weeks	Two hours, across 8 sessions	90 min online education opened 2 months	20 min, over 3‐week period	Education intervention program ran for 54 days One off education
1.7 Supporting material and resources	None reported	Introduction to OHC protocol and assessment tool Access to OHC products and equipment	Access to OHC products and equipment Availability of OHC protocol and assessment tool Access to specialist dental services	Access to OHC products and equipment Incorporating OHC education into new staff orientation Oral care champions	Access to OHC products and equipment

### 4.3 Recipients of Intervention

The main recipients of the oral healthcare education interventions were registered nurses, clinical support workers, and nursing students (Ab Malik et al. 2017; Brady et al. 2011, 2020; Smith et al. 2017). A single study targeted the multi‐disciplinary team (Letsos et al. 2013) while another study reported that allied health staff were invited to training as well; however, only nurses participated in surveys (Brady et al. 2011). The number of staff who received oral healthcare education interventions ranged from 50 to 547, while the sample of those who participated in intervention evaluation ranged from 12 to 373.

### 4.4 Development Principles & Design of Oral Healthcare Education Intervention

Two of the five studies provided explicit information about the guiding principles or framework to inform oral healthcare education program design. One study applied the theory of planned behaviour to inform its educational intervention, using its domains to guide content design (Ab Malik et al. 2017). Another study applied the Medical Research Council framework for complex intervention design (Smith et al. 2017). Co‐design incorporating staff focus group feedback and a multidisciplinary workshop was utilised by Smith et al. (2017) and Brady et al. (2020), and Letsos et al. (2013) utilised best practice guidelines and literature to inform their project design.

### 4.5 Content, Delivery and Resources

The content of the oral healthcare education interventions ranged from good oral healthcare principles (Ab Malik et al. 2017), importance of oral healthcare and consequences of poor oral healthcare (Ab Malik et al. 2017), and the nurse's role in oral care for stroke patients (Ab Malik et al. 2017). The ideal context and nature of supporting patient's oral healthcare following stroke (Brady et al. 2011) in addition to hands‐on training with oral healthcare tools and products was employed in three studies to consolidate theoretical learning (Brady et al. 2011; Letsos et al. 2013; Smith et al. 2017). Two studies utilised web‐based or Computer‐assisted learning platforms to deliver the oral healthcare education program (Ab Malik et al. 2017; Brady et al. 2020) and two studies facilitated face‐to‐face training (Brady et al. 2011; Letsos et al. 2013). Reported lengths of education sessions ranged from 20 to 120 min (Brady et al. 2011, 2020; Letsos et al. 2013). Training sessions were generally singular, but in one instance, training was repeated over eight sessions (Brady et al. 2011). Overall intervention implementation periods reported by three studies ranged from 3 weeks to 2 months (Brady et al. 2020; Letsos et al. 2013; Smith et al. 2017). Most studies incorporated a range of resources and support materials to complement or consolidate oral healthcare education (Brady et al. 2011, 2020; Letsos et al. 2013; Smith et al. 2017). Staff access to a suite of resources such as oral healthcare tools, products, equipment, and support staff, including oral care champions, were components of the complex interventions to support oral healthcare training. In the QI project by Letsos et al. 2013, oral healthcare education was built into new staff orientation.

### 4.6 Effectiveness, Acceptability and Feasibility of Oral Healthcare Education Interventions

Oral healthcare effectiveness was assessed using measures pertaining to service levels, staff impact, and patient outcomes. The effectiveness of oral healthcare education on the provision of oral healthcare was determined by examining knowledge, attitudes, confidence, and practice adherence, while the evaluation of the interventions was determined by feasibility and acceptability. Table 3 provides a summary of intervention effectiveness, acceptability, and feasibility.

**TABLE 3:** Effectiveness, acceptability and feasibility of oral healthcare education interventions.

	Ab Malik et al. (2017)	Brady et al. (2011)	Brady et al. (2020)	Letsos et al. (2013)	Smith et al. (2017)
Outcome measures	Staff questionnaires used TPB guided outcomes of: ○ General intentions ○ Attitudes ○ Subjective norms ○ Perceived behaviour control	Pre and post‐intervention staff knowledge and attitudes using an adapted questionnaire Post‐intervention auditing of documented MDT referral for OCH support Feasibility via semi‐structured interviews	Pre and post‐intervention staff knowledge and attitudes via questionnaire Auditing of documented OHC assessment, care plans and MDT referral for OCH support Feasibility in web‐based training engagement	Pre‐intervention questionnaire to assess baseline knowledge Pre and post‐intervention bedside audits of OHC products Audit of documented of OHC assessment, oral care plans, provision, and MDT OHC collaboration	Feasibility of intervention measured as time to complete training and be registered as component post ‘hands‐on’ practical sessions Acceptability, barriers and adequacy of OHC education assessed post‐implementation via focus groups
Knowledge	Significant difference in changes in knowledge scores between test and control group at 6 month (*p* = 0.001)	Significant difference after training, in 4 of 25 items: Efficiency of toothbrush size (*p* < 0.001) Tooth decay risk due to lack of calcium (*p* < 0.001) Mouth swab as an alternative to toothbrush for cleaning patient's teeth (*p* = 0.022) Patients with dry mouth tend to get less decay (*p* < 0.001)	No change in OHC knowledge after training No difference between RNs and other nursing staff in scores	No post‐intervention OHC knowledge measured. However, pre‐survey showed 3 knowledge gap areas Uncertainty of oral care provision frequency Incorrect OHC product usage Uncertainty around frequency and timing of oral health assessments, with responses ranging from: within the first hour of admission (39.1%); within 24 h of admission (47.8%); within 48 h (4.3%) and unsure (8.7%).	Web‐based material was informative and improved knowledge of oral anatomy which then supported staff in undertaking the oral hygiene protocol during dental simulation
Attitudes	Significant difference in changes in attitude score between test and control group at 1 month (*p* = 0.04) and 6 months (*p* = 0.009)	Significant difference after training, in 1 of 12 items: I believe my own teeth should last me throughout my life (*p* = 0.0064)	No change in OHC attitudes after training No difference between RNs and other nursing staff in scores	n/a	n/a
Confidence	n/a	n/a	n/a	n/a	Simulation exercise in hands‐on training sessions helped with confidence to deliver OHC protocol in ward environment
Practice adherence	n/a	No documented referrals to MDT for OHC specific management	Poor staff adherence to documentation of OHC assessment and plans, with 2 of the 4 sites identify transition to paper to electronic records during the study, altering how staff could record OHC plans An increase from 6 pre‐intervention to 10 referrals dental specialists during intervention, but no report of referrals post‐intervention	96.4% compliance of oral health assessment tool usage within 24 h of admission in a bedside audit Proper stocking of recommended OHC products via audit No reporting of whether there is improvement in oral care provision frequency, despite it being identified as a knowledge gap in pre‐intervention survey Evidence of MDT collaboration specific to patient OHC needs occurring daily And documented OHC recommendations in patient medical charts	
Feasibility & acceptability	n/a		Due to OHC training being optional, there was variability in staff engagement across sites, ranging from 41.1% to 94.7% of all staff	n/a	A total of 54 days for 50 staff to complete web‐based training and be registered as competent post hands‐on training and assessment OHC education found to be acceptable and easy to use by most participants Would have liked supplementary classroom‐based teaching Simulation exercise did not depict real‐life difficulties encountered

Staff questionnaires were the most frequently used instrument (Ab Malik et al. 2017; Brady et al. 2011, 2020; Letsos et al. 2013) and were adapted from, or used in, previous research. Documentation adherence (Brady et al. 2011, 2020; Letsos et al. 2013), rate of oral healthcare referrals/collaboration with the multidisciplinary (MDT) team (Brady et al. 2011, 2020; Letsos et al. 2013), knowledge (Letsos et al. 2013), attitudes (Brady et al. 2011, 2020), general intentions, and social norms Ab Malik et al. (2017) were measured. One study conducted pre‐and post‐intervention bedside audits to evaluate correct oral healthcare product placement (Brady et al. 2020). Three studies evaluated intervention implementation feasibility (Brady et al. 2011, 2020; Smith et al. 2017), and Smith et al. (2017) also reported acceptability, knowledge, and confidence.

#### 4.6.1 Knowledge, Attitudes, and Confidence

There were minimal improvements in oral healthcare knowledge, and confidence in three of four studies (Ab Malik et al. 2017; Brady et al. 2011, 2020; Smith et al. 2017). In the mixed methods study by Brady et al. (2011), only 4 (16%) knowledge items improved on completion of staff education; efficiency of toothbrush size (*p* < 0.001), tooth decay risk due to lack of calcium (*p* < 0.001), mouth swab as an alternative to toothbrush for cleaning patient's teeth (*p* = 0.022), and patients with a dry mouth tend to get less decay (*p* < 0.001). One (8.3%) attitude item “I believe my own teeth should last me throughout my life” improved after training (*p* = 0.0064) (Brady et al. 2011) and the subsequent study by Brady et al. (2020) showed no difference in nursing oral healthcare attitudes or knowledge. Letsos et al. (2013) reported pre‐intervention knowledge gaps in oral care provision frequency, oral healthcare product usage and frequency and timing of oral health assessments, but failed to conduct a post‐intervention survey to ascertain practice change.

#### 4.6.2 Oral Healthcare Practice Adherence

In most studies, oral healthcare education did not improve oral healthcare practice adherence. Poor staff adherence to documentation of oral healthcare assessment and plans (Brady et al. 2020) and an absence of reporting the frequency of oral health care provision Letsos et al. (2013) featured in study findings. Referrals to multi‐disciplinary teams (MDT) for oral healthcare specific management were variable (Brady et al. 2011, 2020) with the exception of the study by (Letsos et al. 2013) that described evidence of MDT collaboration specific to patient oral healthcare needs occurring daily.

#### 4.6.3 Feasibility & Acceptability of Oral Healthcare Education Interventions

A single feasibility assessment indicated that the time to undertake the web‐based and hands‐on training for 50 staff was 54 days (Smith et al. 2017). In the study by Brady et al. (2020), training was optional and staff engagement varied across sites, ranging from 41.1% to 94.7%. Participants in the sequential mixed method study by (Smith et al. 2017) claimed the intervention could have benefited from supplemental classroom‐based teaching, and the simulation exercise examples did not reflect real‐life situations. Issues such as logging into web‐based training and interruptions due to ward‐related issues were also discussed as barriers (Smith et al. 2017).

## 5 Discussion

Current stroke clinical practice guidelines identify stroke clinicians (particularly nurses) as key persons to optimal oral healthcare provision among stroke survivors (Intercollegiate Stroke Working Party 2016; Stroke Foundation, 2022; Stroke Foundation of New Zealand and New Zealand Guidelines Group 2010). Stroke clinicians have the potential to assist in the oral healthcare transition of stroke survivors from the acute context to a community setting and could contribute to a reduction in oral health‐related complications, with the appropriate training. This scoping review identified the characteristics, gaps, and areas for improvement in existing educational oral healthcare interventions for stroke clinicians. A key finding from this review was the heterogeneity and complexity of interventions. Only one intervention was a standalone educational program (Ab Malik et al. 2017), whereas the other studies included education as part of a larger suite where referral pathways, assessment tools, or oral health products were also included. The development of varied and complex oral healthcare interventions may reflect the acute needs of stroke survivors, where individuals may have loss in cognitive, memory, and motor function (Go et al. 2013; Hochstenbach et al. 1998). Consequently, many individuals may not be able to adequately self‐care, necessitating additional tools or persons to support oral healthcare (Go et al. 2013; Hochstenbach et al. 1998).

Adapting guidelines to the local context is an important step in the knowledge‐to‐action cycle when implementing clinical practice guidelines and may result in a complex intervention (Margaret et al. 2010). However, the necessary detail and description around the interventions' development and their underpinning frameworks were not explicitly articulated in the studies. Only one mentioned the use of a theoretical framework (Ab Malik et al. 2017), while two others incorporated codesign participatory methods (Brady et al. 2020; Smith et al. 2017), although the codesign processes were not transparent. Furthermore, high levels of adherence in practice and engagement in the training were not reported. While most studies were assessed as moderate to high quality, the lack of explicit detail from the included studies around the codesign process demonstrates a need for more rigorous research and evaluation methods in this aspect (Slattery et al. 2020). An approach that could be undertaken may involve a process similarly employed by Lievesley et al. (2022), who described their method in combining both experienced‐based codesign processes (Robert 2013) and the behaviour change wheel (Michie et al. 2011) to inform an oral health intervention among stroke survivors. The development and implementation of such interventions would also need to involve change agents, such as health management staff and supervisors, to identify and address systemic barriers to compliance and change (Cardoso et al. 2023).

The few studies that explored intervention acceptability and feasibility observed that the participants viewed the intervention as acceptable and beneficial to their clinical practice. As expected, a combination of theoretical and practical education was perceived to be the most ideal for participants. Research on learner‐centred pedagogies, where the learner may be an active participant and engage in hands‐on learning, has demonstrated that this pedagogical approach is typically well‐received by both educators and students (Bremner et al. 2022). Furthermore, other research supports the use of relevant simulation‐based learning to result in better learning outcomes (Delisle et al. 2019). Given that the provision of oral care as well as education and referrals among stroke survivors is a practical task, incorporating appropriate simulations into education interventions would be a logical step, irrespective of whether the theoretical content is delivered online or face‐to‐face.

The findings from this review indicate that the educational interventions had some improvement in stroke clinicians' oral health knowledge, attitudes, confidence, and subjective norms; however, none of the studies measured a significant change in clinical practice post implementation of the intervention. While one quality improvement project (Letsos et al. 2013) identified over 90% compliance with the oral health assessment tool after the intervention, there were no comparator assessments undertaken. This gap in knowledge‐to‐practice was discussed in focus group findings from Smith et al. (2017) where participants appreciated the knowledge but admitted that it did not result in changes in “real‐life” practice. The paucity of translation into practice may be reflected in the low levels of response rate across the studies, highlighting a need for implementation and engagement strategies to be administered alongside educational interventions.

Some of the interventions were designed as a pilot, which acknowledges that the process in developing an effective education programme may require further modification. Evidence‐based implementation strategies may need to be administered alongside education interventions to improve changes in professional practice. Systematic approaches to implementation are needed at both the initial and ongoing stages of the intervention to test mechanisms of change, measure effectiveness of multifaceted strategies on whether they improve clinical outcomes, and improve the tracking of these implementation strategies (Powell et al. 2019). In addition to educational meetings and distribution of materials, dissemination and implementation strategies could include local opinion leaders, or champions; audit and feedback; or ongoing reminders in clinical practice (Brady et al. 2011; Letsos et al. 2013; Medves et al. 2010).

To improve clinical oral health outcomes, such as reducing aspiration pneumonia among stroke survivors, there is the need to develop an adequate oral healthcare education intervention for stroke clinicians that effectively demonstrates changes in oral healthcare practice. This intervention would need to be underpinned by a robust theoretical framework and methodology to improve the processes around developing an educational intervention. Future studies would also need to enhance transparency by providing more detail on the development and characteristics of the intervention, particularly given that most of the interventions in this review were complex. Measuring research outcomes would also need to be directed by validated instruments to determine the intervention's effectiveness.

## 6 Conclusions

Existing standalone and complex oral healthcare education interventions have shown some potential in building capacity, competency, and confidence in stroke clinicians to provide oral healthcare but have yet to demonstrate changes in clinical practice and patient outcomes. The findings and gaps in the literature highlight the need for future interventions to incorporate a robust theoretical framework and methodology, consider the development of an education programme as part of a larger, complex intervention, and to incorporate both theoretical and relevant, practical action‐based simulations. Future studies in this area should also employ implementation strategies alongside the dissemination of the programme to improve translation and long‐term sustainment of the intervention.

## Author Contributions

A.G., C.F. and S.A. conceived and designed the study. L.L. and B.T. developed the search strategy and conducted the literature search and initial screening assessment. The second screening of the articles was reviewed by A.G., L.L., B.T. and C.F. and A.G., S.W. and P.S. undertook the quality assessment. A.G., L.L., C.F., R.W., S.A. and A.K. completed data synthesis and interpretations and prepared the first draft of the manuscript. All authors provided input into versions of the manuscript and read and approved the final manuscript.

## Conflicts of Interest

The authors declare no conflicts of interest.

## Supporting information


Data S1.

Data S2.

## Data Availability

Data sharing is not applicable to this study as no new data was created. The data for this study is openly available from the databases CINAHL, Medline (Ovid), ProQuest, PubMed, SCOPUS, Cochrane library as well as grey literature.
