# Supplementary figures and images for: The Characteristics and Effectiveness of Oral Healthcare Education Interventions for Stroke Clinicians: A Scoping Review
Source: J Clin Nurs. 2025 Apr 28;34(9):3473–88. doi: 10.1111/jocn.17795 (PMC12340753; doi:10.1111/jocn.17795)

**Additional File 2: Search Strategy**

Database: Ovid MEDLINE(R) ALL

Search Strategy:


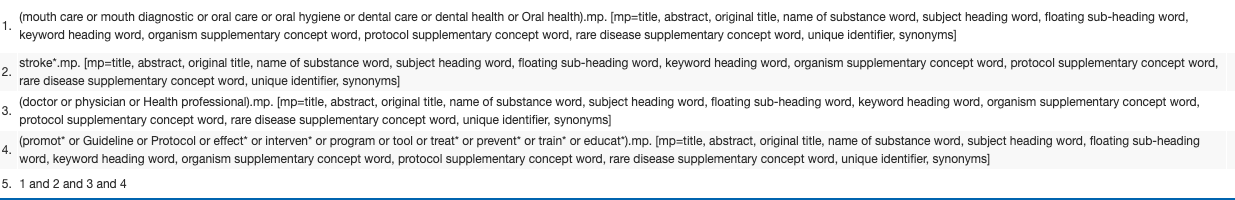

Supplement: Supplementary file 2 — Data S2. [file JOCN-34-3473-s002.docx]
